# Onset of Buccal Pumping in Catshark Embryos: How Breathing Develops in the Egg Capsule

**DOI:** 10.1371/journal.pone.0109504

**Published:** 2014-10-20

**Authors:** Taketeru Tomita, Masaru Nakamura, Keiichi Sato, Hiroko Takaoka, Minoru Toda, Junro Kawauchi, Kazuhiro Nakaya

**Affiliations:** 1 Hokkaido University Museum, Hakodate, Hokkaido, Japan; 2 Okinawa Churashima Research Center, Okinawa Churashima Foundation, Motobu, Okinawa, Japan; 3 Okinawa Churaumi Aquarium, Motobu, Okinawa, Japan; 4 Graduate School of Fisheries Science, Hokkaido University, Hakodate, Hokkaido, Japan; 5 Hokkaido University, Hakodate, Hokkaido, Japan; Inner Mongolia University, China

## Abstract

Respiration in fishes involves buccal pumping, which is characterized by the generation of nearly continuous water flow over the gills because of the rhythmic expansion/compression of the pharyngeal cavity. This mechanism is achieved by the functions of the vascular, skeletal, and muscular systems. However, the process by which the embryo establishes the mechanism remains a mystery. Morphological and kinematical observations on captive cloudy catsharks, *Scyliorhinus torazame*, have suggested that the embryo starts buccal pumping just before the respiratory slits open on the egg capsule. During the pre-opening period, the embryo acquires oxygen mainly via the external gill filaments. After slit opening, respiration of the embryo involves buccal pumping to pass water over the “internal gills.” The onset of buccal pumping accompanies four morphological changes: (1) regression of the external gill filaments, (2) development of blood vessels within the “internal gills,” (3) completion of the development of hyoid skeletal and muscular elements, and (4) development of the oral valve. A previous study showed that buccal pumping allows the embryo to actively regulate oxygen intake by changing the pumping frequency. Thus, establishment of buccal pumping in the egg capsule is probably important for embryo survival in the unstable oxygen environment of the egg capsule after slit opening.

## Introduction

Chondrichthyes (sharks, batoids, and chimaeroids) are unique among fishes in producing exceptionally large embryos (e.g. [1], [2], [3]). Such embryos potentially have difficulty in acquiring enough oxygen [4]. In general, small embryos, like those of bony fishes, use the entire body surface as the main respiratory organ [5]. However, for large embryos with a small surface area-to-volume ratio, gas diffusion through the body surface may be insufficient, and some special adaptations may be required.

In chondrichthyan embryos, the external gill filaments have been proposed as the organ that meets an embryo’s oxygen demands [4], [6]. This structure is characterized by extremely elongated gill lamellae extending outside of the gill slits. In some species, the external gill filaments are almost as long as the embryo itself [7]. External gill filaments are estimated to supply at least 50% of the entire oxygen requirement in skates [4].

It is widely accepted that in chondrichthyan embryos, the external gill filaments are an important respiratory organ [8], [9], [10], but the processes underlying the switch from the embryonic respiratory system to the adult system remain a mystery. One of the keys to solving this mystery is to clarify the early development of “buccal pumping,” which involves the rhythmic compression/expansion of the pharyngeal region. Many fishes use this mechanism for generating a water current over the gills (e.g. [11]). It is known that the late-stage embryo of the small-spotted catshark, *Scyliorhinus canicula*, breathes by buccal pumping [12], [13]. Studies on buccal pumping behavior in adult small-spotted catsharks have revealed that this behavior is accomplished by the action of several respiratory valves and the skeletal and muscular elements of the pharynx [14], [15]. However, the process in which these anatomical structures develop in association with the establishment of buccal pumping is poorly understood.

The purpose of the present study was two-fold: first, to describe the early development of the anatomical structures associated with buccal pumping, and second, to clarify the early ontogeny of the embryonic respiratory system in the cloudy catshark, *Scyliorhinus torazame*.

## Materials and Methods

### Fish

Egg capsules were laid by captive, adult cloudy catsharks, *S. torazame*, in 2012 and 2013 at the Okinawa Churaumi Aquarium. The eggs were incubated in the tanks at the Marine Research Center of the University of Ryukyu (Okinawa, Japan). Water temperature was maintained at 15°C for the first month and 16±1°C thereafter. For morphological and kinematical observations, we sampled a total of 35 embryos from the incubation tank. The embryos used for the observations ranged from 16.7 to 84.9 mm in total length (TL), and from 51 days to 132 days in incubation period after parturition. For comparison, we also examined a dead adult cloudy catshark that had been collected off Hakodate, Hokkaido, Japan in 2013.

### External morphology and kinematics

To observe embryos inside egg capsules, an egg capsule was placed in a plastic tray, which was then illuminated from below. Water inside the tray was constantly renewed by seawater from the incubation tank. We recorded the presence/absence of buccal pumping. Among the individuals that demonstrate buccal pumping, we measured the duration of 50 mouth opening/closing cycles for 51.5, 53.5, 56.8, and 84.9-mm TL embryos, and also measured the duration of 10 mouth opening/closing cycles for 39.0 and 44.2-mm TL embryos. Measurement was performed three times per individual and the average was calculated.

The egg capsules were then removed for all 35 embryos and observed under an optimal microscope (KeyenceVHX-1000; Keyence Co., Osaka, Japan). The lengths of gill filaments on the first gill slits were measured, and mean length was calculated for each individual. The presence/absence of an “oral valve” (the respiratory valve just behind the upper jaw [16], [17]) was also observed.

**Figure 1:**
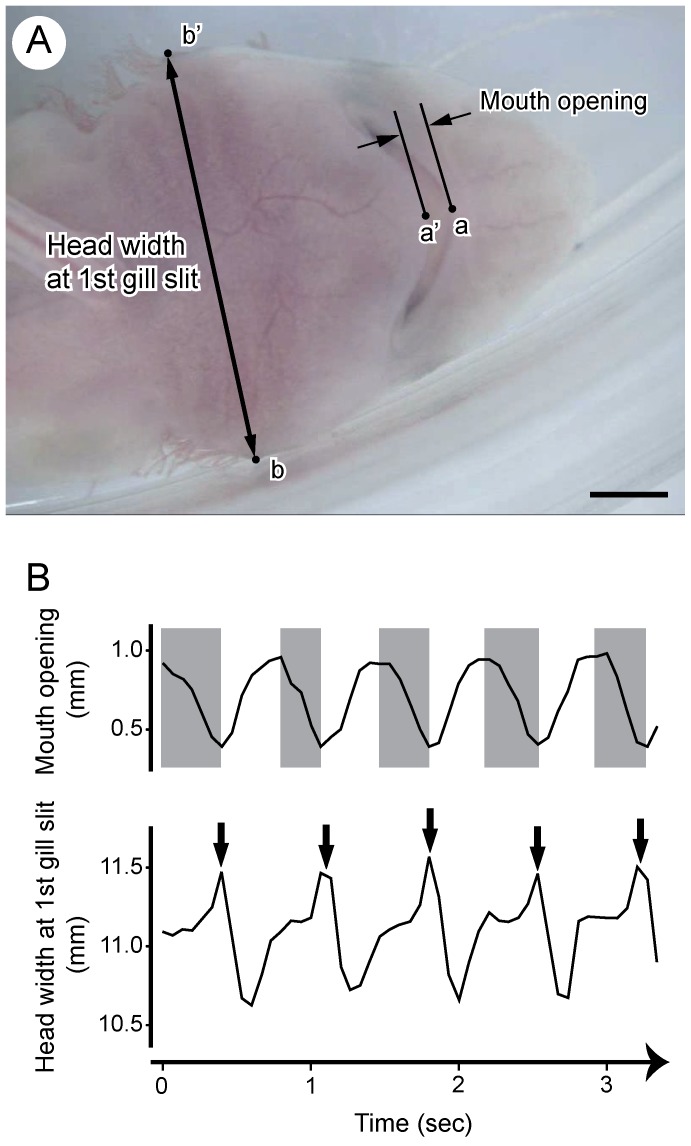
Buccal pumping of 58.6 mm TL embryo of the cloudy catshark (*Scyliorhinus torazame*) in ventral view. A. Digitized points for quantifying the movement of mouth and pharyngeal regions. Descriptions of each measurement point (a, a′, b, and b′) are shown in Material and method section. Scale bar = 2 mm. B. Changes of amount of mouth opening (above) and head width at the first gill slit (below) over time. Gray areas in above figure indicate the duration of mouth closing that represents the compression phase of oro-pharyngeal cavity. Arrows in below figure indicate the timing of the opening of the first gill slit.

In addition, video footage was acquired from 5 individuals (53.3, 56.8, 58.6, 75.8, and 84.9-mm TL embryos) for quantifying the patterns of buccal pumping, using the optimal microscope (KeyenceVHX-1000; Keyence Co.). The recording time for each individual ranged from 1 to 3 minutes. For quantifying the patterns of buccal pumping in each embryo, still images were captured at 66.7-ms intervals from the first 10 s of each video footage using movie editing software (KMplayer 2.9.4.1.1435; Jelsoft Enterprises Ltd., Pangbourne, UK). After obtaining the sequence of still images, the coordinates were digitized from each still image using the coordinate-measuring tool in ImageJ software (US National Institutes of Health, Bethesda, MD, USA). Coordinates acquired for each image were: 1) anterior tip of the upper jaw and anterior tip of the lower jaw (Fig. 1, points a and a′), and 2) the gill margin of the first gill opening on either side of the head (Fig. 1, points b and b′). From these coordinates, the degree of mouth opening (distance between points a and a′) and head width at the first gill slit (distance between points b and b′) were calculated for each image. In order to estimate the time lag between oropharyngeal compression and the subsequent opening of the first gill silts, we also calculated the time delay (ms) at the maximum head width at the first gill slit after the minimum gape time for each buccal pumping cycle (duration between the first and next minimum gape).

After these observations were made, all 35 specimens were euthanized with phenoxyethanol and then fixed in Bouin’s solution for anatomical analysis (see below). The procedure for fixation with Bouin’s solution was based on the method described in [18]. Maintenance and handling of fish and all experiments were conducted in strict accordance with the Hokkaido University Guide for Care and Use of Laboratory Animals (http://www.hokudai.ac.jp/research/ethics/animal/), which stipulates that procedures for the care and use of lower vertebrates (fishes and amphibians) are to be conducted with the same considerations for animal care and welfare as those for higher vertebrates (reptiles, birds, and mammals). As stipulated in the Guide, however, the requirement for approval from the University of Hokkaido Institutional Animal Care and Use Committee, which is necessary for protocols involving the use higher vertebrates, is waived for protocols involving the use of lower vertebrates.

### Histological thin sections

A series of histological thin sections were taken from seven specimens (23.2, 25.8, 27.2, 29.2, 32.8, 39.8, and 53.5-mm TL embryos). The head area, including the gills, was serially sectioned at 7 µm, almost perpendicular to the body axis. The sections were stained with Delafield’s hematoxylin and eosin, and observed under a digital microscope (BX53; Olympus) at Churashima Research Center (Okinawa). For the 25.8-mm TL embryo, thin sections were stained with a periodic acid-Schiff (PAS) staining kit (1.01646.0001; Merck KGaA) and were also counterstained with hematoxylin for detection of aldehyde and mucosubstances, in accordance with the procedure of the kit. Using this staining method, the basal membrane was stained purple. We measured the vascular diameter within gill lamella from histological thin sections. Vascular diameter was estimated from the length of minor axis of vascular cross sections.

Three-dimensional (3D) reconstructions of pharyngeal, skeletal, and muscular elements were generated from serial images of the thin sections for four specimens (27.2, 29.2, 32.8, and 39.8-mm TL embryos). Firstly, each slice was photographed using a digital camera (DP73; Olympus) mounted on the microscope. Secondly, cross sections of skeletal and muscular structures were colored for each image using Adobe Illustrator CS4 (Adobe Systems Inc.), and new serial images were created. Several thin sections were considerably deformed through the preparation of thin sections (e.g., some parts were separated and had moved from their original positions). For such sections, separated parts were moved back to the original place based on the adjacent anterior and posterior slices. Thirdly, 3D reconstruction was performed from the colored serial images using Image J. The final illustrations were obtained using the open source 3D editing software, Blender 2.69.

## Results

### Buccal pumping and lateral head movements

The smallest individual that carried out buccal pumping was 39.0 mm TL. The buccal pumping frequencies were observed to be 4.0±4.1, 11.4±4.2, 51.0±7.8, 58.1±1.9, 51.3±6.0, and 57.4±6.6 s.d. mouth-closings per minute in 39.0-, 44.2-, 51.5-, 53.5-, 56.8-, and 84.9-mm TL specimens, respectively. The pumping frequency of the three large specimens (≥51.5-mm TL specimens) was significantly higher than that of the two smaller specimens (39.0- and 44.2-mm TL specimens) (p = 0.017, t-test).

During buccal pumping, the first gill slits opened just after the duration of mouth closing in all specimens used for the kinematical analysis (Fig. 1, Movie S1). The time lags between oropharyngeal compression (time at minimum gape) and the subsequent opening of the first gill slits (time at maximum head width at the first gill slit) were 16.7±70.3, 0.0±44.4, 15.4±29.2, 73.3±37.8, and 26.7±39.9 s.d. ms in 53.3-, 56.8-, 58.6-, 75.8-, and 84.9-mm TL embryos, respectively. These time lags were very small in comparison with the duration of a single pumping cycle (1.7±8.4, 0.1±4.9, 2.0±3.9, 0.08±0.04, and 5.4±8.0 s.d.% of a single pumping cycle in 53.3-, 56.8-, 58.6-, 75.8-, and 84.9-mm TL embryos, respectively). This indicates that the water in the branchial cavity was eliminated via the gill slit during or just after oropharyngeal compression.

Strong lateral head movement (Movie S2), caused by fanning of the caudal fin, was observed in all specimens except for 75.1, 75.8, and 84.9-mm TL specimens. These three specimens were tightly packed in the egg case, and their head movements were spatially restricted.

### External gill filament length

**Figure 2:**
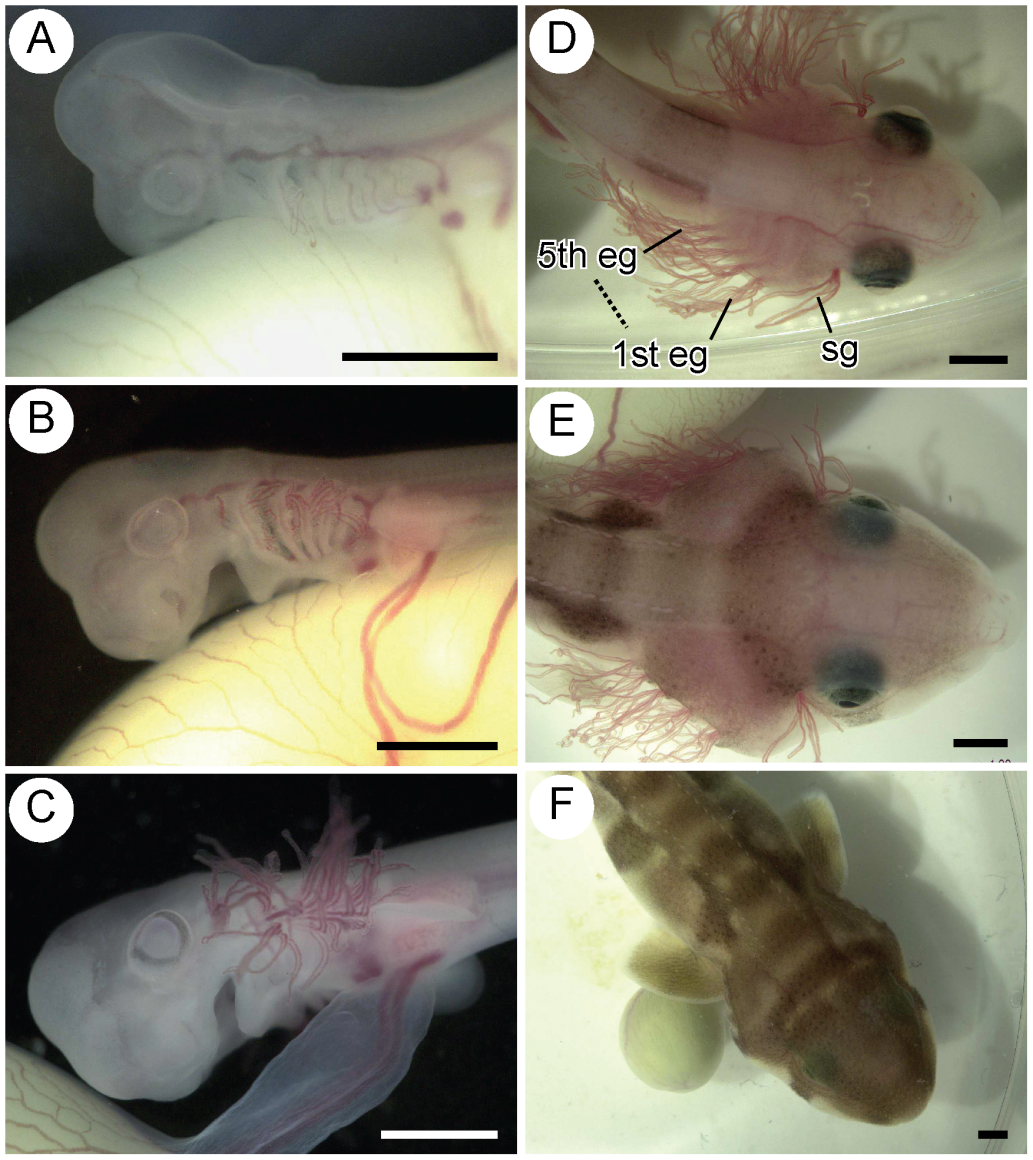
Morphological change of head and pharyngeal regions through ontogeny. A. 19.2 mm TL. B. 21.9 mm TL. C. 27.2 mm TL. D. 43.6 mm TL. E. 53.3 mm TL. F. 75.1 mm TL. Scale bars = 2 mm. eg = external gill filaments, sg = spiracular gill filaments.

**Figure 3:**
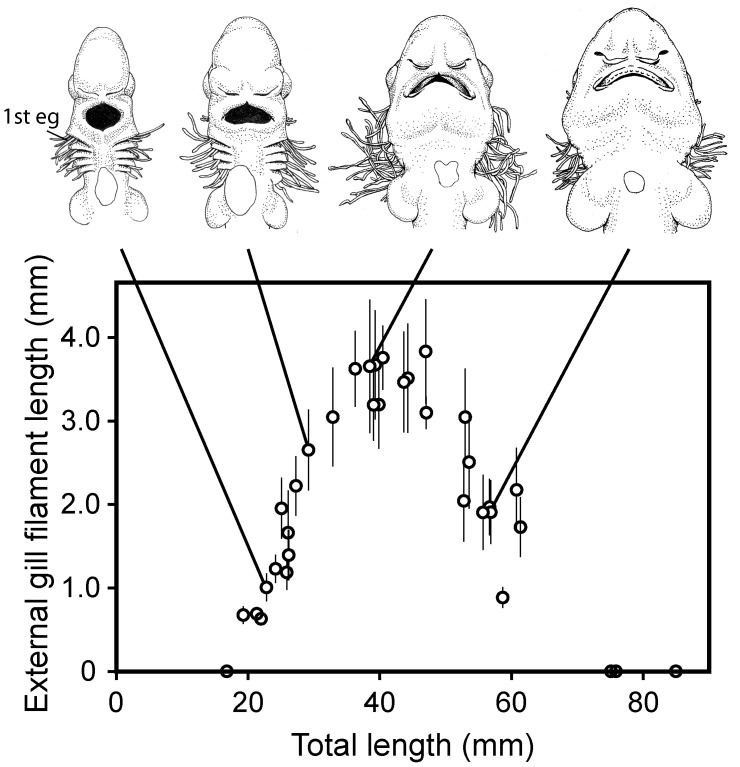
Length (±standard deviation) of the external gill filaments on the first gill slit for 35 embryos. The above figures represent the ventral view of the head and pharyngeal region. 1st eg = external gill filaments on the first gill slit.

The change in the length of the external gill filaments is shown in Fig. 2 and Fig. 3. The filaments first appeared when the embryo was about 20 mm TL. Filaments developed in sequence from the first to the fifth gill slits, with the spiracular gill filaments developing last. Filaments reached their maximum length (3.2–3.8 mm) when the embryo was around 40 mm TL. Thereafter, external gill filaments regressed and had completely disappeared by the time the embryo was around 65–75 mm TL.

### Development of the skeletal and muscular system of the hyoid arch

**Figure 4:**
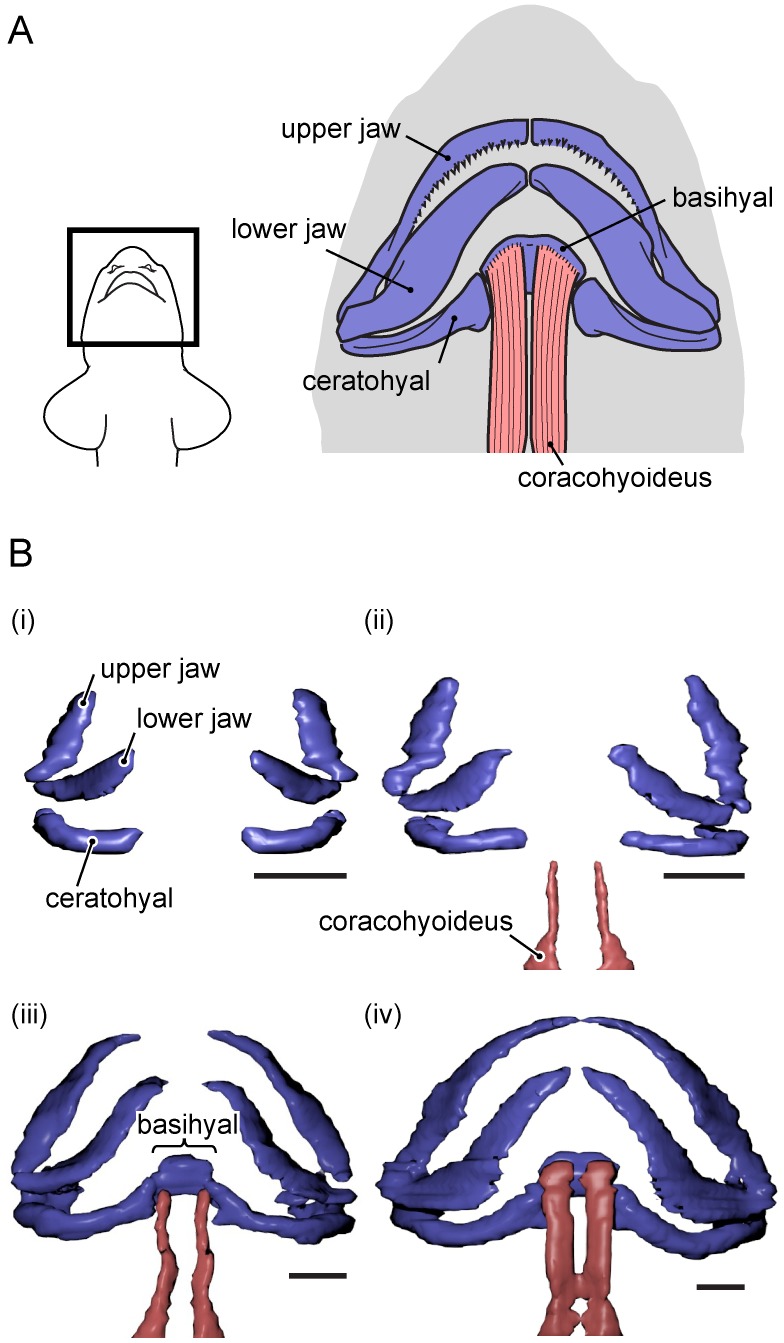
Development of pharyngeal skeletal and muscular elements in the pharyngeal region. A. Schematic diagram of the pharyngeal skeletal and muscular elements of the adult cloudy catshark. B. 3-dimensional reconstructions of pharyngeal skeletal and muscular elements made from histological thin sections. Ventral view of the jaw and pharyngeal skeletal elements (blue) and the hyoid retraction muscle (coracohyoideus; red) in 27.2 mm TL (i), 29.2 mm TL (ii), 32.8 mm TL (iii), and 39.8 mm TL (iv) embryos. Scale bars = 500 µm.

The hyoid arch is the second visceral arch and is located just behind the mandibular arch (jaws) [19]. The hyoid arch of adult sharks is composed of three elements (from ventral to dorsal): a basihyal, a ceratohyal, and a hyomandibula, with the latter connected to the otic region of the cranium (Fig. 4A). The coracohyoideus muscle is attached to the ventral surface of the basihyal cartilage. Contraction of this muscle retracts the hyoid arch in a posteroventral direction, resulting in expansion of the pharyngeal cavity.

The development of hyoid skeletal and muscular elements is shown in Fig. 4B. In the 27.2-mm TL embryo, the posterior region of the ceratohyal had formed (Fig. 4B–i). In the 29.2-mm TL embryo, the ceratohyal slightly extended anteriorly (Fig. 4B–ii). A pair of coracohyoideus muscles had developed, but their anterior ends were not yet attached to the hyoid skeletal elements. In the 32.8-mm TL embryo, the basihyal cartilage had formed and the hyoid skeletal apparatus was complete (Fig. 4B–iii), with the coracohyoideus muscle attached to the lateral-most side of the basihyal cartilage. In the 39.8-mm TL embryo, the coracohyoideus muscles were more robust, and their anterior ends covered the entire ventral surface of the basihyal cartilage (Fig. 4B–iv).

### Development of anterior-side gill lamellae

**Figure 5:**
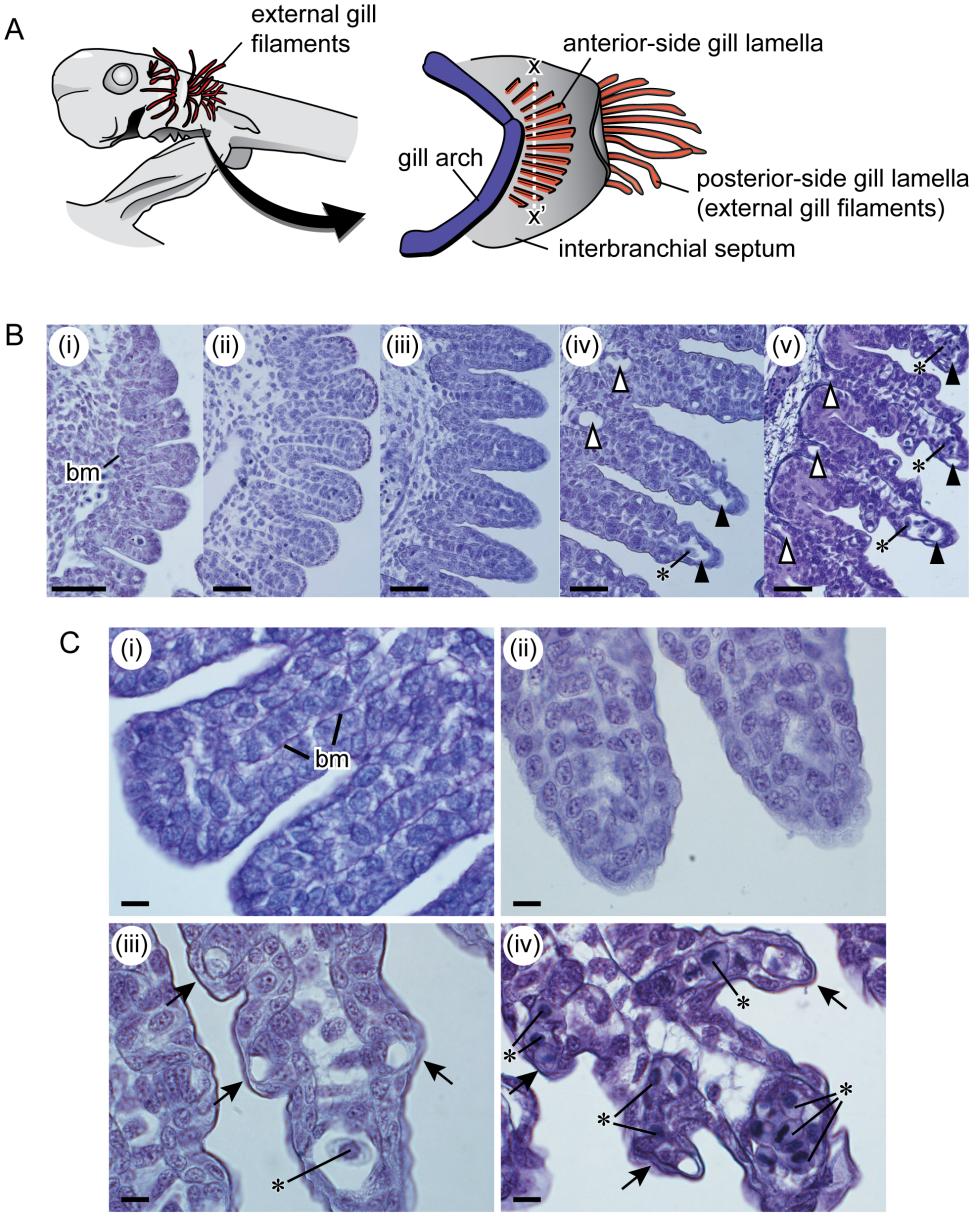
Development of anterior-side gill lamellae. A. Lateral view of the head region of a cloudy catshark embryo (left), and schematic diagram of the embryonic gill structure (right). Posterior-side gill lamellae (external gill filaments) are elongated and extend out of the gill slit, whereas lamellae on the anterior side develop and remain inside the gill slit. B. Histological thin sections of the anterior-side gill lamellae on the first gill arches of 23.2 mm TL (i), 27.2 mm TL (ii), 32.8 mm TL (iii), 39.8 mm TL (iv), and 53.5 mm TL (v) embryos. Sections were made along the dotted line (x–x′) in figure of A, right side. Black triangles represent efferent lamellar arteries; white triangle, afferent lamellar arteries. BM represents basal membrane. Asterisks (*) in the efferent and afferent lamellar arteries represent red blood cells. The images in (iii), (iv) and (v) are horizontally flipped to allow for easier comparison. Scale bar = 50 µm. C. Close-up view of the anterior-side gill lamellas of 25.8 mm TL (i), 32.8 mm TL (ii), 39.8 mm TL (iii), and 53.5 mm TL (iv) embryos. Basement membrane (bm) was stained in purple with PAS staining in narrow space beneath the single- or double-layered epithelium cells (i). Secondary gill lamellas (arrows) and blood cells (*) in the blood vessels present within the gill lamella in (iii) and (iv). Scale bars = 10 µm.

The fish gill was composed of many gill lamellae, some on the anterior and some on the posterior face of each interbranchial septum (Fig. 5A). A previous study showed that the posterior- and anterior-side lamellae arise through different processes (e.g. [20]). Those on the posterior side are formed in the early embryonic period as filamentous structures extending outside the gill slits, and are known as external gill filaments. (In the late-stage embryo, the external gill filaments regress and recede into the gill slit.) On the other hand, anterior-side lamellae start to develop in middle-stage embryos. In contrast to posterior-side lamellae, lamellae on the anterior side develop inside the gill slits, and are not exposed throughout development.

The developmental process of anterior-side lamellae is shown in Fig. 5B. In the 23.2 and 25.8-mm TL embryos, epithelial ridges were present on the lateral surface of the visceral arches, forming the anterior-side lamellae (Fig. 5B–i, Fig. 5C–i). The surface of these ridges consisted of a single or double layer of cells. These cell layers were supported by the basal membrane (colored purple with PAS staining in Fig. 5C–i). In the 27.2-mm TL embryo, the ridges were more prominent (Fig. 5B–ii) and the surface consisted of a double cell layer, which is the general condition of adult elasmobranchs. In the 32.8-mm TL embryo, a small channel (the future efferent lamellar artery) was present within the lamella (Fig. 5B–iii, 5C–ii), but its diameter (ca. 5 µm) was less than that of red blood cells (c.a. 15 µm), restricting their passage through the channel (Fig. 5C–ii). In the 39.8-mm TL embryo, the efferent and afferent lamellar arteries were developed (Fig. 5B–iv, 5C–iii) and their diameters (ca. 30 µm, and therefore greater than those of red blood cells) included many red blood cells. Secondary gill lamellae had started to develop. In the 53.5-mm TL embryo, secondary gill lamellae were well developed, and additional channels had formed within the secondary lamellae (Fig. 5B–v, 5C–iv). These channels were filled with red blood cells.

### Development of the oral valve

**Figure 6:**
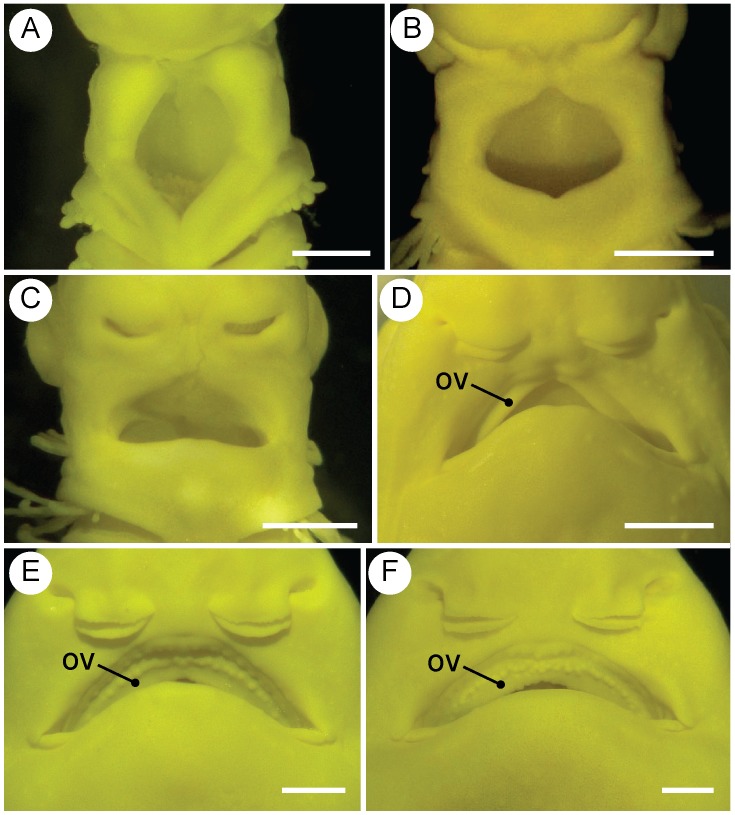
Development of the oral valve (OV). Ventral views of the mouth region of 16.7 mm TL (A), 23.2 mm TL (B), 26.0 mm TL (C), 38.4 mm TL (D), 46.9 mm TL (E), and 53.3 mm TL (F) embryos. Scale bars = 0.5 mm in (A) and (B). Scale bars = 1.0 mm in (C), (D), (E), and (F).

The smallest individual that had an oral valve was 32.8 mm TL. All individuals larger than this had well-developed oral valves. Given that the 26.0-mm TL shark lacked an oral valve, the valve probably develops when sharks are between 26.0 and 32.8 mm TL (Fig. 6).

## Discussion

The present study revealed that the gill and pharyngeal structures of the cloudy catshark embryos drastically change in association with the onset of buccal pumping. Cloudy catsharks start buccal pumping at ca. 40 mm TL, which is roughly half of the hatching size. Prior to the onset of buccal pumping, four morphological changes occur: 1) the external gill filaments start to regress; 2) blood vessels develop in the anterior-side gill lamellae, which indicates that these lamellae become functional around this time; 3) the hyoid retraction muscle (coracohyoideus) covers the entire ventral surface of the anterior-most part of hyoid arch (basihyal), forming a strong connection between the hyoid retraction muscle and hyoid arch – this connection is known to allow the retraction of the hyoid apparatus for creating the negative pressure during buccal pumping in adult sharks [14], [15]; 4) the oral valve develops, which is probably important for generating the nearly unidirectional respiratory current during buccal pumping, as this structure is used for preventing water from exiting through the mouth during compression of the buccal cavity in adult sharks [11], [16], [17].

The onset of buccal pumping probably reflects the shift of a catshark’s major respiratory organ from the external gill filaments to the gill lamellae located inside the gill slits (“internal gills”). Before the onset of buccal pumping, respiration of the embryos likely largely relies on the external gill filaments. The lateral head movements of the embryos during this period may contribute to efficient gas exchange at the surface of these filaments. When buccal pumping begins, the embryo starts to take water in through the mouth and expel it through the gill slits. This novel water pathway causes water to pass over the surface of the “internal gills” for efficient gas exchange here. This hypothesis is consistent with our finding that the anterior-side gill lamellae, which are located inside the gill slits, become functional slightly prior to the onset of buccal pumping. When the embryo starts buccal pumping, it is likely that the embryo acquires oxygen both via the external gill filaments and the “internal gills.” In contrast, pre-hatched embryos probably obtain oxygen solely through the “internal gills,” because such embryos have completely lost the external gill filaments. Regression of the external gill filaments and the start of buccal pumping were also reported in the late embryonic stage of *Scyliorhinus canicula* and *Heterodontus portjacksoni* [6], [12], suggesting that similar ontogenetic processes are shared by other egg-laying species.

The present study also suggests that the onset of buccal pumping may be an adaptation to the unstable oxygen environment within the egg capsule after the opening of its respiratory slits. Late-stage embryos are known to renew the water in the egg capsule through the small slits that appear on both sides of the egg capsule (e.g. [13], [21], [22], [23], [24]). Our data showed that these slits open slightly before the onset of buccal pumping (ca. 35 mm TL). A previous study showed that the oxygen environment inside the egg capsule becomes quite unstable after the opening of slits. During the pre-opening period, the oxygen tension in the egg capsule is almost constant. Conversely, during the post-opening period, oxygen tension in the capsule widely fluctuates [25]. Under these unstable oxygen conditions after the opening of the slits, buccal pumping is possibly a more suitable mechanism rather than that of the external gill filaments, because the embryo can actively regulate oxygen intake by changing pumping frequency. In fact, late-stage embryos of the small-spotted catshark, *S. canicula*, can change the pumping frequency depending on oxygen tension [13], [25]. This ability would also be necessary for post-hatched sharks to adjust their oxygen intake in various oxygen environments or during various activities.

## Supporting Information

Movie S1 **Buccal pumping of a cloudy catshark embryo (58.6 mm in total length).**
(MP4)

Movie S2 **Lateral head movement of a cloudy catshark embryo (38.4 mm in total length).**
(MP4)
